# Inheritance of self- and graft-incompatibility traits in an F_1_ apricot progeny

**DOI:** 10.1371/journal.pone.0216371

**Published:** 2019-05-09

**Authors:** Patricia Irisarri, Tatyana Zhebentyayeva, Pilar Errea, Ana Pina

**Affiliations:** 1 Unidad de Hortofruticultura, Centro de Investigación y Tecnología Agroalimentaria de Aragón (CITA), Instituto Agroalimentario de Aragón—IA2 (CITA-Universidad de Zaragoza), Zaragoza, Spain; 2 The Schatz Center for Tree Molecular Genetics, Department of Ecosystem Sciences and Management, The Pennsylvania State University, University Park, Pennsylvania, United States of America; Huazhong Agriculture University, CHINA

## Abstract

Floral self-incompatibility affecting yearly yield in a weather-dependent manner and graft incompatibility affecting longevity of mature trees are two important traits for apricot production. However, genetic control of graft compatibility and relationship between these traits are unknown. Here, we analyzed its inheritance in an F_1_ apricot progeny from a cross between self- and graft- incompatible and self- and graft-compatible cultivars. Hybrid individuals were genotyped for establishing self-incompatibility status and grafted on the plum rootstock ‘Marianna 2624’. Phenotyping of graft incompatibility was done at two time points, one month and one year after grafting. Anatomical (necrotic layer, bark and wood discontinuity for two consecutive years) and cytomorphological (cell proliferation, cell arrangement and cell shape one month after grafting) characteristics related to graft compatibility displayed continuous variation within the progeny, suggesting a polygenic inheritance. Using the Pearson correlation test, strong and significant correlations were detected between anatomical and cytomorphological traits that may reduce the number of characters for screening genotypes or progenies for graft compatibility in segregating crosses. Furthermore, no correlation existed between self- and graft incompatibility traits suggesting that they are independent inheritance traits. Hence, screening an extended hybrid population is required for pyramiding these traits in breeding programs.

## Introduction

Grafting is largely used in the production of vegetable and fruit-bearing crops to increase uniformity, vigor and adaptation to biotic and abiotic stresses. Compatibility of rootstock and scion plays crucial role for establishing highly efficient root systems through grafting [1, 2]. However, this trait varies significantly even between closely related species, that necessitates evaluation of compatibility before grafting specific scion genotype into rootstock [3, 4]. For stone fruit industry that heavily relies on vegetative propagated cultivars (i.e. individual genotypes) via grafting, long-term vitality of union between rootstock and scion is crucial. Nevertheless, incompatibility not always becomes apparent immediately after grafting. It may take several years to manifest failure with establishing graft-union that leads to major economic losses to growers and nurseries. In addition, the significant delay in the appearance of incompatibility symptoms renders the evaluation and transfer of new fruit tree genotypes to industry time-consuming, expensive and laborious [5, 6].

Apricot is the third most important species of the stone fruit crops with a worldwide production of approximately 3.88 million tons in 2016 [7]. Apricot production is limited by the soil conditions of the different growing areas, such as heavy, calcareous soils with iron chlorosis and waterlogging problems, very frequent in the Spanish Mediterranean area (Aragon, Murcia and Valencia) and suffers from incomplete compatibility. In order to solve these problems, a wide array of species have been used, such as apricot, peach and plum seedlings or interspecific hybrids as Marianna 2624 (*P*. *cerasifera* L. × *P*. *munsoniana* L.). Nevertheless, graft incompatibility between most popular *Prunus* rootstocks and apricot cultivars is one of the major problems for rootstock usage and improvement. Failure in producing long-leaving healthy grafts greatly affects the range of available *Prunus* rootstocks for apricot cultivation [8]. Incompatible graft combinations show weakness of the graft interface and breakage of the rootstock/scion union, which are typical symptoms of the localized type of incompatibility [5, 6, 9]. The late manifestation of this type of incompatibility has made that many studies focused on the early physiological and molecular mechanisms underlying graft union formation and graft (in)-compatibility in apricot grafted onto different *Prunus* rootstocks [10–13]. A lot of research was done on anatomical, physiological and molecular factors useful for forecasting compatible rootstock-scion combinations on an industrial scale [13, 14]. The genotypes of both rootstock and scion play an important role in rootstock-scion interaction affecting the success of graft union formation and the quality of vascular connections at the graft union [15–17]. Recently, several studies have shown that molecular changes may be involved in varied pattern of performance in compatible/incompatible combinations [10, 18–20]. Changes in transcriptomic profiles have been found between compatible and incompatible graft combinations in different species (Litchi, apricot, *Citrus*, melon) [12, 20, 21]. In these studies, differentially expressed genes (DEGs) related to stress response, auxin and signal transduction at early stage of grafting might determine graft compatibility or incompatibility. Transcriptomic analyses are particularly complicated in woody plants because grafting is done on dormant wood so that a graft-union formation coincides with the reactivation of the cambium in the spring [22]. It is not easy to separate transcripts which are specific to the wood development, the graft formation and incompatibility responses. Despite these difficulties, a large number of metabolic pathways (such as phenylpropanoid pathway, cell wall biosynthesis or oxidative stress, genes related to auxin signaling) have been associated with this agronomic trait and could be considered as potentially involved in physiological failure in graft incompatible rootstock-scion combinations [10, 18, 20, 23, 24], suggesting complex genetic control of incompatibility reaction.

Floral self-incompatibility (SI) is another trait affecting yield in apricot. Many fruit species in the *Rosaceae* (apricot, apple, pear, plum, sweet cherry, Japanese plum, and almond) exhibit typical gametophytic self-incompatibility (SI) controlled by multi-allelic S-locus [25, 26]. SI is a not desirable trait for apricot cultivation since it limits productivity and breeding efficiency because of low fruit set [27]. In contrast to graft incompatibility, a large body of studies have been undertaking to understand the mechanism of self-incompatibility [26, 28, 29]. Self-incompatibility response in the *Rosaceae* may involve several signaling factors, activation of MAPKs, calcium levels, ROS or cell death. ROS accumulation and activation of apoptosis signaling pathways occurring during pollen-pistil interactions in the *Rosaceae* could be similar to the same biological processes that take place during the scion/rootstock interaction. However, whether the two traits (self- and graft-incompatibility) are genetically dependent or not is still unknown.

Although the genetic control mechanisms of some agronomic traits in fruit trees (tree development, flowering and ripening, chill requirement, self-incompatibility, pest and disease resistance, fruit quality and fruit production) are well understood [30], there is almost no information about the genetic basis underlying graft compatibility. This fact is due to the difficulties in evaluating the trait and lack of adequate model system in woody trees. In order to quantify grafting success of a particular scion/rootstock combination many plants need to be grafted and this is a logistical problem for genetic studies involving large plant populations, and especially if an incompatibility phenotype is hidden for several years after grafting [9, 15]. Furthermore, in fruit trees, it could take many years for the plants to produce sufficient quantity of woody stems/buds for large-scale experiments. Hence, inheritance mode for graft incompatibility in woody plants is missing, and probability of selection a “perfect rootstock”, i.e. graft-compatible and self-compatible rootstock variety with stable seed production, is largely unknown. For this reason, here we report results on histopathological manifestation of graft incompatibility and its inheritance in fruit trees. As a model system, we used an apricot progeny from a cross between self- and graft- incompatible and self- and graft-compatible cultivars. Hybrid individuals were genotyped for establishing self-incompatibility status and grafted on the plum rootstock ‘Marianna 2624’. Phenotyping of graft incompatibility was done at two time points, one month and one year after grafting.

## Material and methods

### Plant material

The plant material for this study included a population of 156 apricot progeny from a cross between the Spanish cultivar ‘Moniqui’ (female parent, graft incompatible) and the French cultivar ‘Paviot’ (male parent, graft compatible) [9]. Pollination was performed in 2011 at CITA of Aragon (Spain). To avoid pollinating insects, female tree was isolated in a 0.8-mm mesh cage before bloom. In caged trees, the flowers were cross-pollinated using a thin paintbrush every other day until all flowers had opened [31]. Anthers were isolated from flowers at the balloon stage and placed on paper at room temperature for 24 h until their dehiscence. Pollen was then sieved through a 0.26-mm mesh and stored at –20 °C [32]. In spring 2012, germinated seeds were planted in pots and grown in a greenhouse. In January, three branches (with length of around 20 cm and a diameter of 5 mm) were picked from each seedling and placed in a growth chamber under controlled conditions (4°C). In the spring of 2013 and 2014, 92 F_1_ individuals and the parents were grafted onto the plum rootstock ‘Marianna 2624’ (*P*. *cerasifera* L. *× P*. *munsoniana* L.) (1−year−old plants with a diameter of 1 cm). Twenty grafts were performed by chip-budding for each individual seedling and characterized for graft compatibility twice, in one month (spring 2014) and one year after grafting (spring 2015).

### Anatomical characterization

Grafts were cut 5 cm above and below the union one year after grafting. The internal characterization of the graft union was carried out through a longitudinal cut at the graft area with a DW876 saw (Dewalt, Italy). Anatomy on the surface of the union was observed using a stereomicroscope (Wild Heerbrugg, Switzerland) equipped with a digital imaging system through a DC300 camera (Leica mycrosystems, Germany). The evaluation was performed as followed: necrotic line, woody and bark discontinuity (scored between 0 = absence and 5 = maximum) [33].

### Microscope observations

Grafts were cut 5 cm above and below the union one month after grafting, fixed in ethanol (95%)/acetic acid 3:1 (v/v) over 24 h, and then transferred to ethanol (70%) at 4°C for conservation [34]. Longitudinal free-hand sections were obtained to the surface of the graft in all unions with a high-profile microtome blade. To evaluate the contact surface, longitudinal sections were transferred into a Petri dish and stained for 30 seconds with cellulose-specific dye solution 0.07% (w/v) calcofluor in distilled water. Sections were then observed using an Olympus BH2-RFCA fluorescence microscope equipped with a digital imaging system through a DC300 camera (Leica microsystems, Germany). Three replicates were tested for every graft combination. Assignment phenotypic scores to individuals was based on cell pattern, i.e. cell arrangement, cell shape and cell proliferation at the graft interface. In total progeny were assigned to 5 phenotypic classes for cell arrangement and cell proliferation depending on the level of compatibility with ‘Marianna 2624’ rootstock—from 0 (absence, graft incompatible plants) to maximum 5—(graft compatible plants). The cell shape was classified from 1 (small and round cells, graft incompatible plants) to 4 (elongated cells distributed in rows, graft compatible plants).

### Self-incompatibility phenotyping

Self-incompatibility phenotypes were assigned to progeny using molecular markers linked to the trait following published protocol with few modifications [35]. Genomic DNA was extracted from young leaf samples following the protocol described by Hormaza [36]. The DNA concentration was measured using Nanodrop 1000 (Thermo Fisher Scientific, USA) and diluted to a final concentration of 10 ng/μl. Two primer sets, the AS1 and PRU-C4R, designed by Tao et al. [37] were used for amplification with the following parameters: an initial denaturation of 3 min at 94°C, followed by 34 cycles of 30 s at 94°C, 45 s at 55°C and 1 min at 72°C, and a final extension of 10 min at 72°C. The PCR products were separated on 1% agarose gel and visualized by staining with ethidium bromide in a gel-doc equipment (UV transilluminator Gel Doc 2000 (BIO-RAD Hercules, USA).

### Phenotypic statistical analysis

All statistical analyses were performed using SPSS 21.0 statistical software (SPSS Inc., USA.). The distribution of the seedling population for each trait was represented in frequency histograms using mean values of the two years of the study for each individual. The normality of each trait distribution was evaluated by the Kolmogorov–Smirnov and the Shapiro-Wilks tests implemented in the SPSS package. Traits with normal distribution linked to graft incompatibility were analyzed by ANOVA considering individuals and years as independent factors. Bivariate correlations between and within the different traits and years were calculated using the Pearson correlation coefficients. Hierarchical cluster analysis of the quantitative phenotypic data was carried out using the squared Euclidian distance combined with the average linkage clustering method. A dendrogram was constructed to evaluate the homogeneity of the clusters using the inter-linkage group method.

## Results and discussion

### Inheritance of a graft incompatibility in F_1_ progeny

**Fig 1:**
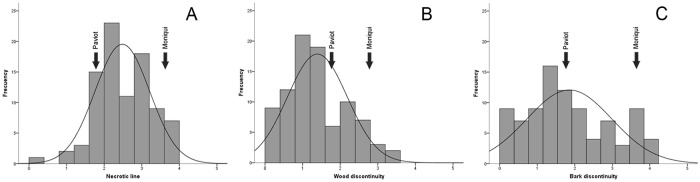
Distribution of proportion of the 92 seedlings of ‘Moniqui’ x ‘Paviot’ F_1_ apricot progeny grafted on ‘Marianna 2624’ evaluated for different graft incompatibility traits. Necrotic line (A), wood (B) and bark discontinuity (C) one year after grafting for the years 2014 and 2015.

Labor-intensive and time-consuming process of evaluating graft compatibility is a major drawback in phenotyping multiple progeny. We evaluated different anatomical parameters related to graft incompatibility (necrotic line, wood and bark discontinuity) in an apricot progeny one year after grafting in 2014 and 2015. Based on the two years of evaluation, we observed variability and noticeable segregation in the progeny for traits associated with graft incompatibility (Fig 1). The distribution of the seedling population for the necrotic line spread in 68.54% of the 92 descendants phenotyped, and a normal distribution of this trait was observed in the population. Some of the individuals had smaller values than ‘Paviot’ (23.59%) and higher (7.86%) than ‘Moniqui’ (Fig 1A). Regarding wood discontinuity, mean values of descendants showed significant differences one year after grafting, with mean values between 0 and 4.72 this parameter had intermediate values between the progenitors in 25.84% of descendants. However, a high number of hybrid plants showed transgressed values (out of the range of the parents) with 68.54% of the descendants with lower values than the male parent ‘Paviot’ and 5.62% with values higher than the female parent ‘Moniqui’ (Fig 1B). In relation to bark discontinuity, 42.70% of the descendants had lower values that the progenitor ‘Paviot’ and 4.49% had values higher than ‘Moniqui’. The population had a normal distribution, but bark discontinuity in many descendants was less than that in progenitors (Fig 1C). Normal distribution phenotypic classes indicated that all traits linked to graft incompatibility (necrotic line, wood and bark discontinuity) likely have a polygenic nature. In addition, the presence of values out of the range of the parents suggest the influence of genetic background on the expression of these polygenic traits, which are potentially inherited from a grandparent (parent of a parent). It should be taken into account that the transmission of graft incompatibility is important for optimizing configuration crosses and improving efficiency of both scion and rootstock breeding programs [6].

**Fig 2:**
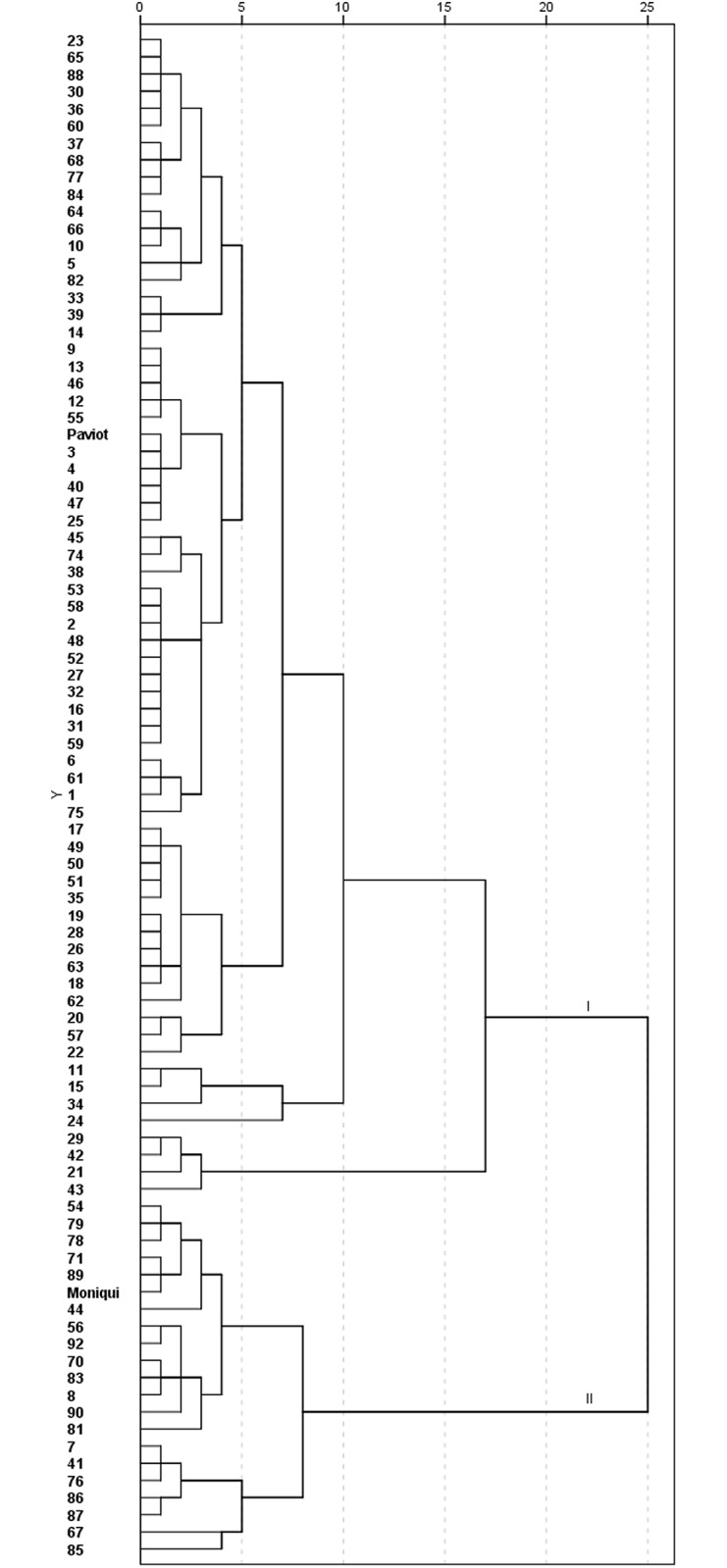
Cluster analysis of the F_1_ apricot progeny ‘Moniqui’ x ‘Paviot’ grafted on the rootstock ‘Marianna 2624’ based on the phenotypic parameters necrotic line, wood and bark discontinuity one year after grafting.

We performed a progeny classification based on quantitative phenotypic data for necrotic line, wood and bark discontinuity. Individuals plants that have missing values for traits at least one year were excluded from analysis. Remaining individuals with complete phenotypic datasets were grouped into two main clusters according to degree of incompatibility. As expected, male parent ‘Paviot’ was grouped with compatible progeny in Cluster I while female parent ‘Moniqui’ was clustered with incompatible progeny in Cluster II (Fig 2). In total, 75.28% and 24.72% of descendants were found in Cluster I (compatible) and Class II (incompatible) respectively (Fig 2). Therefore, the segregation ratio was 3:1 that implies a dominant gene effect in case of monogenic trait. This segregation ratio was in agreement with our previous study with lower number of F1 apricot individuals evaluated based on one year of anatomical observations [38]. However, it is unknown how many genes are involved in genetic control of graft incompatibility. Many traits of agricultural significance exhibit quantitative inheritance, which is often the result of multiple genes of minor effect, such as pest and disease resistance, fruit quality and fruit production [30, 39]. High number of hybrid plants with transgressed values for graft-associated traits indicate that graft compatibility most likely is a complex agronomic trait under multigenic control.

### Cytomorphological pattern associated with graft incompatibility in the progeny

**Fig 3:**
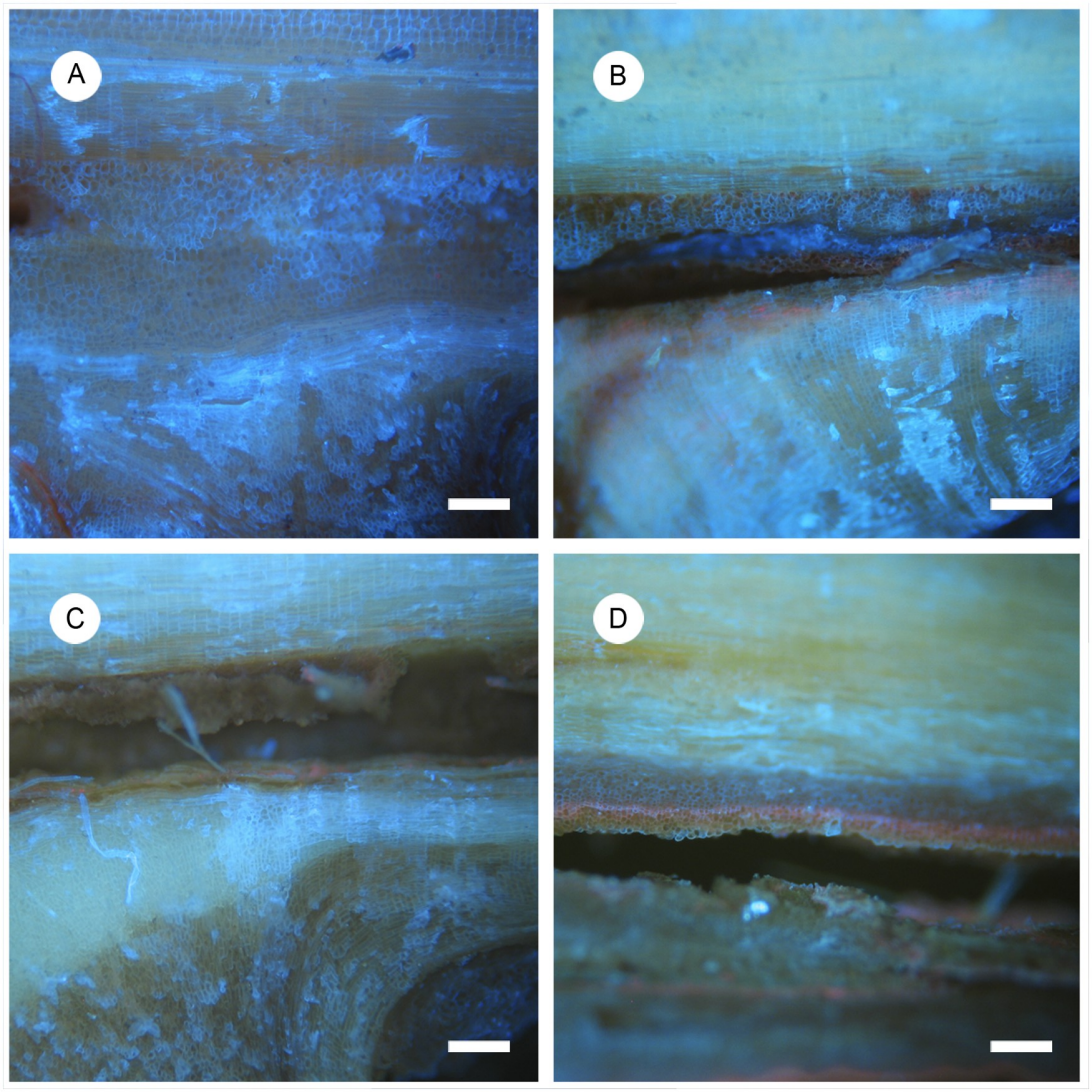
Cytomorphological patterns associated with graft incompatibility in the F_1_ progeny grafted on the rootstock ‘Marianna 2624’ one month after grafting. Longitudinal sections were stained with calcofluor and phenotypic scores were based on cell proliferation, cell arrangement and cell shape at the graft interface. (A) Graft interface completely filled with ordered and round cells and new vascular connections established, (B) graft union partially filled with less defined and ordered cells, (C) groups of unordered and poorly defined cells at the graft interface and (D) Not cell proliferation at the graft interface.

**Fig 4:**
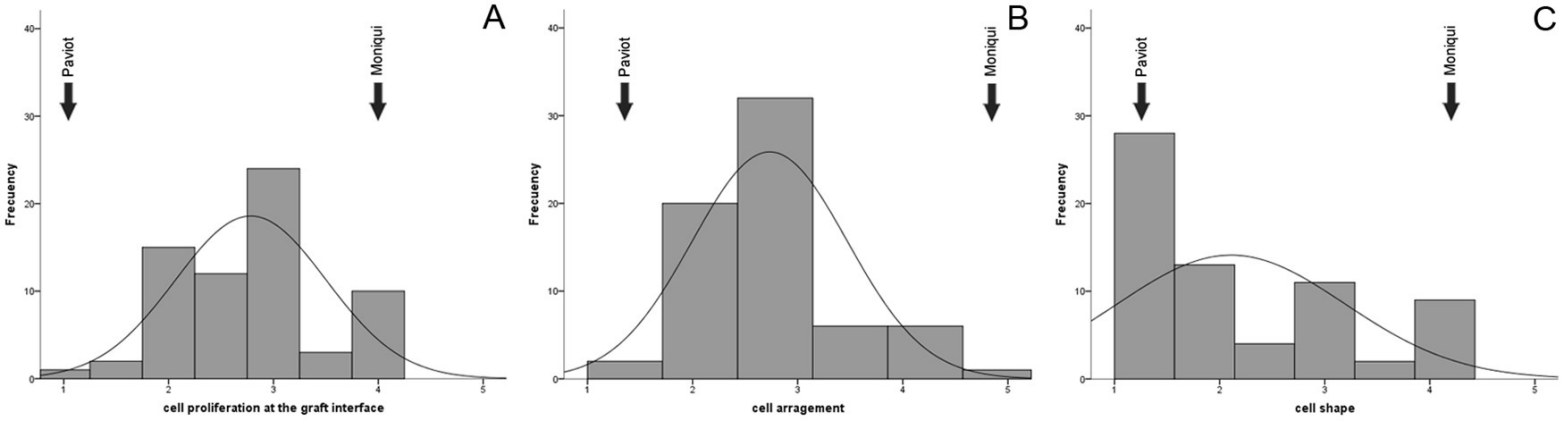
Distribution of proportion of 65 seedlings of ‘Moniqui’ × ‘Paviot’ F_1_ apricot progeny grafted on ‘Marianna 2624’ based on cytomorphological observations. Cell proliferation at the graft interface (A), cell arrangement (B) and cell shape (C) one month after grafting.

Previous studies indicated that the content and nature of the callus cells involved in the first step of graft formation might play an important role in triggering the response that leads to the formation of a strong and successful union [40, 41]. Cell proliferation, cell arrangement and cell shape were observed in 65 apricot seedlings grafted on the rootstock ‘Marianna 2624’ and evaluated at two time points, one month and one year after grafting. Parental genotypes grafted on ‘Marianna 2624’ were used as compatible and incompatible controls. Compatible grafts exhibited an organized and homogeneous cell arrangement at the contact surface, strongly stained with calcofluor (Fig 3A and 3B). In incompatible scion-rootstock combinations we observed a disorganized arrangement in some areas of the contact surface, showing no additional development (Fig 3C and 3D). All the characteristics of the descendants spread between the compatible and incompatible parents. We observed a normal distribution of these traits in hybrid population (Fig 4). At first time point, one month after grafting, descendants were morphologically intermediate if compare them with phenotypic extremes represented by parents. So, all early cellular signals related to graft compatibility were transmitted to the descendants. Grafts union formation in same individuals at one month after grafting and one year after grafting significantly correlated based on morphological changes at the graft interface. We assume that morphological differences at the cellular level reflect graft compatibility/incompatibility not only in the Mo × Pa background. Therefore, cytological inspection of graft union at one month after grafting provides an early test for determining the affinity of grafted seedlings. Potentially, morphological pattern of graft union reported here can predict graft compatible phenotype in other progenies and in new cultivars to be released to the market.

### Pearson correlations between the phenotypic traits

Correlations were found among most agronomic traits linked to graft incompatibility during the two years of the study (2014 and 2015). Significant positive Pearson correlations existed between the phenotypic parameters observed in the F1 progeny, necrotic layer and wood discontinuity (r = 0.474**) and necrotic line and bark discontinuity (r = 0.565**) in 2014. The same trend was observed in the year 2015 (Table 1). Hence, these three parameters are highly correlated and play an important role in the development of the graft union and the establishment of vascular connections. A large body of studies have reported that localized graft incompatibility is characterized by anatomical alterations at the graft union area that does not prevent the initial growth of the tree during the first few years [6, 16]. In fact, some studies reported that in compatible unions, the necrotic layer disappears at the moment of cell division in correspondence with the callus formation while, in incompatible grafting (as in Arabidopsis/tomato grafts), the persistence of necrotic layer seems to directly or indirectly inhibit the vascular tissue differentiation, thus blocking full vascular graft union formation between the two graft partners [42]. In addition, Pearson correlations showed a significant strong correlation between cell proliferation and cell arrangement (0,802**), cell shape and cell proliferation (0,521**), as well as cell arrangement and cell shape (0,573**), all of which were significant with p < 0.005 (Table 2). Recent findings on the molecular mechanisms of graft union formation and graft incompatibility in some plant species have provided new insights into rootstock-scion interaction. Different studies revealed transcripts or proteins differentially expressed or accumulated at the graft interface during graft union formation related to signal transduction, auxin transportation, secondary metabolism, cell cycle, phloem and xylem development, wound response, and cell wall synthesis [22, 24]. Differentially expressed genes (DEGs) related to stress response, auxin and signal transduction at early stage of grafting might determine graft compatibility or incompatibility [18, 21, 43].

**Table 1:** Pearson correlation coefficients of the phenotypic parameters necrotic line, wood and bark discontinuity in the 92 seedlings of ‘Moniqui’ X ‘Paviot’ (MXP) F1 apricot progeny grafted on ‘Marianna 2624’ one year after grafting during the years 2014 and 2015.

	necrotic_2014	wood_2014	bark_2014	necrotic_2015	wood_2015	bark_2015	Self-incompatibility
necrotic_2014	1	0,481**	0,565**	0,648**	0,490**	0,488**	-0,970
wood_2014	0,481**	1	0,649**	0,527**	0,686**	0,587**	-0,095
bark_2014	0,565**	0,649**	1	0,480**	0,552**	0,855**	-0,050
necrotic_2015	0,648**	0,527**	0,480**	1	0,703**	0,653**	-0,170
wood_2015	0,490**	0,686**	0,552**	0,703**	1	0,698**	-0,086
bark_2015	0,488**	0,587**	0,855**	0,653**	0,698**	1	0,009
Self-incompatibility	-0,970	-0,095	-0,050	-0,170	-0,086	0,009	1

** Correlation significant at the 0.01 level (bilateral)

**Table 2:** Pearson correlation coefficients for different phenotypic parameters at early stages of development. Cell proliferation, cell arrangement and cell shape were evaluated in 65 seedlings of ‘Moniqui’ X ‘Paviot’ (MXP) F1 apricot progeny grafted on ‘Marianna 2624’ one month after grafting.

	Cell proliferation	Cell arrangement	Cell shape
Cell proliferation	1	0,802**	0,521**
Cell arrangement	0,802**	1	0,573**
Cell shape	0,521**	0,573**	1

** Correlation significant at the 0.01 level (bilateral)

On the other hand, necrotic line, wood and bark discontinuity showed good correlations with a reduced influence of year. All the phenotypic parameters showed a positive and significant correlation between years. The average values between years were r = 0,648 for necrotic line, 0.686 for wood discontinuity and 0.885 for bark discontinuity (Table 1). Thus, our results indicated non-significant environmental effect for manifestation graft compatibility in progeny between graft-compatible and graft-incompatible parents. In this regard, inheritance of graft compatibility in F_1_ apricot cross was similar to that for other agronomic traits such as fruit quality related characters (ripening time, skin color) or reproductive phenology [44–46].

The establishment of correlations between anatomical traits may reduce the number of characters for screening genotypes or progenies for graft compatibility. It helps to optimize time and labor required for evaluation of a high number of grafts per individuals in segregating crosses. Based on results presented here, we recommend using our phenotyping approach for phenotyping graft compatibility in other fruit and forest trees. Thus, we propose an earlier anatomical test for evaluation of compatibility between variety industrial rootstocks and new cultivars released to market.

### Self-incompatibility and relationship with graft compatibility

**Fig 5:**
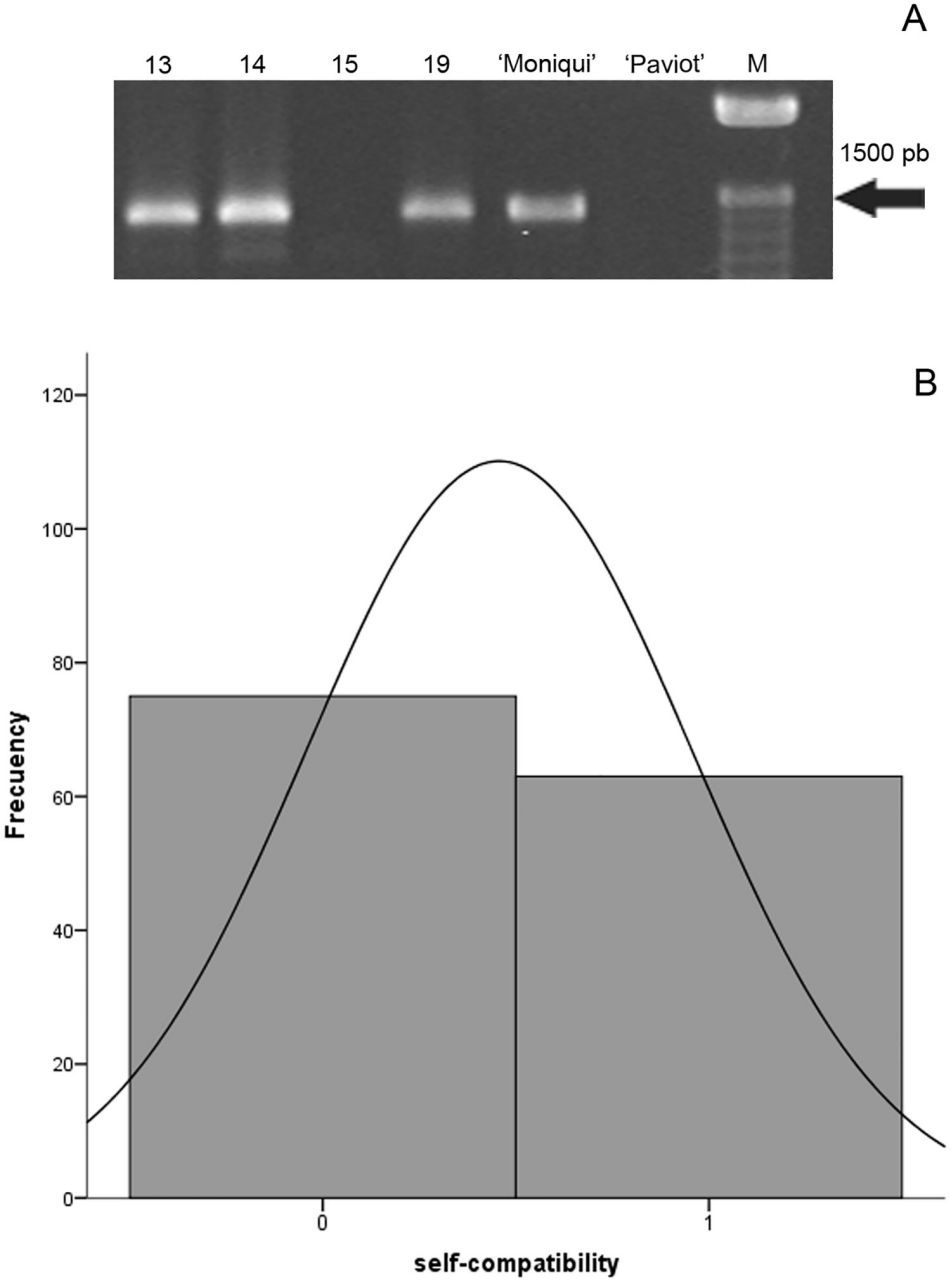
PCR analysis of the self-incompatibility allele in the 156 individuals from the ‘Moniqui’ x ‘Paviot’ apricot population. Amplified fragments were obtained by PCR from genomic DNA using AS1 and Pru-C4R [35] primers. Results from 4 individuals of the ‘Moniqui’ x ‘Paviot’ progeny (13, 14, 15 and 19) and their parents ‘Moniqui’ and ‘Paviot’. Band size was determined by comparison with 100-bp molecular weight marker (M) on the right.

Self-incompatibility is an important genetic mechanism that prevents inbreeding and promotes genetic polymorphism and heterosis in flowering plants. The inheritance of apricot self-incompatibility has been studied in progenies within different programs carried out worldwide to obtain new self-compatible cultivars with good agronomical and commercial quality [27, 35]. However, there are no studies, in which the transmission of both agronomical traits (self- and graft-incompatibility have been examined in the same genetic background. In total, 138 apricot seedlings of the F_1_ cross ‘Moniqui × Paviot’, together with the ‘Moniqui’ and ‘Paviot’ progenitor cultivars, were genotyped for an S-allelic composition using an express PCR test [35, 37]. In parents, presence of the self-incompatibility allele (Si) was consistent with data by Vilanova [35]. Self-incompatible female parent ‘Moniqui’ showed one band around 1.5 kb, whereas DNA of self-compatible male parent ‘Paviot’ did not amplify any band. Consequently, the ‘Mo × Pa’ population segregated for presence-absence amplification product. Based on the PCR genotyping test, 53.6% of the descendants were self-compatible and 46.4% self-incompatible (Fig 5A and 5B). Thus, the segregation ratio was 1:1 (Х^2^ = 1.043 at p<0.01) in agreement with findings by previous researchers [35, 47].

We estimated correlation between two traits, graft incompatibility and self-incompatibility, using the Pearson’s metrics (Table 1). The Pearson correlations between self-incompatibility trait and phenotypic parameters linked to graft incompatibility were negative suggesting that no correlation exist between these traits, therefore, they are genetically independent. Thus, screening large number of progenies independently is required for pyramiding these traits in breeding. A large number of studies have discussed the models for the biochemical mechanism of the S-RNase-based self-incompatibility. A critical breakthrough in understanding of the self-incompatibility signal transduction pathway in *Rosaceae* suggested striking alterations in the mitochondrial structure, disruption of reactive oxygen species (ROS) and induction of DNA degradation in incompatible pollen tubes [26, 48]. All these events are characteristics of programmed cell death (PCD) and biological processes related to PCD and ROS accumulation were also reported related to graft incompatibility response [17, 18, 23, 49]. Particularly, the endomembrane system was pointed out as an important player in pollen-pistil interactions [50, 51]. Similarly, cellular contact must be established to enable the formation of a symplasmic and apoplasmic transport system between graft partners and a novel control factor (signal transmission) of connectivity would reach the graft partner and change its innate rate of communication affecting the graft success [11, 52, 53]. So, it would be not unexpectedly if some overlapping mechanism of incompatibility between both traits appear at the very basic molecular level.

## Concluding remarks

In our study most of the traits linked to graft incompatibility displayed continuous variation within the progeny and reflected the genetic difference between phenotypically contract progenitors, graft compatible ‘Moniqui’ and graft incompatible ‘Paviot’. Hence, patterns of phenotypic variation across progeny most likely reflects a polygenic inheritance of the trait in this cross. Wherein, the necrotic line, discontinuities in the bark and wood, and cell/tissue organization are highly correlated parameters. These morphological characters may reflect important aspects of developing a graft union and establishing vascular connections. Reported histomorphological approach for phenotyping paves a way for genetic approach using linkage genetic mapping and identification quantitative trait loci (QTLs) for graft compatibility in segregating crosses. Development of molecular markers associated with trait for marker-assisted selection (MAS) would be convenient for increasing efficiency of apricot breeding. A scion-rootstock interaction in apricot is currently a well-characterized model system within the *Prunus* genus that can be used for development molecular markers for accurate prediction of graft compatibility between scion and rootstock in other woody plants.
